# HLA Correlates of Long-Term Survival in Vertically Infected HIV-1-Positive Adolescents in Harare, Zimbabwe

**DOI:** 10.1089/aid.2014.0338

**Published:** 2015-05-01

**Authors:** Brittany L. Shepherd, Rashida Ferrand, Shungu Munyati, Samuel Folkard, Kathryn Boyd, Tsitsi Bandason, Sabelle Jallow, Sarah L. Rowland-Jones, Louis-Marie Yindom

**Affiliations:** ^1^ Nuffield Department of Medicine, University of Oxford, Headington, Oxford, United Kingdom.; ^2^ London School of Hygiene and Tropical Medicine, London, United Kingdom.; ^3^ Biomedical Research and Training Institute, Harare, Zimbabwe.

## Abstract

African infants with vertically acquired HIV infection progress rapidly, with only 50% surviving beyond 2 years in the absence of treatment. Despite this high initial mortality, recent reports describe a substantial burden of older children living with untreated vertically acquired HIV infection in Southern Africa. The immunological and genetic factors associated with long-term survival following vertical infection are poorly understood. We performed medium-to-high resolution HLA typing on DNA samples obtained from a cohort of presumed vertically HIV-1-infected children and age-matched uninfected controls in Harare, Zimbabwe. Overall, 93 HLA class I alleles were detected in the study population with a significant enrichment of HLA-C*08:02 and -C*08:04 in the HIV-1-infected long-term survivor group. Conversely, HLA-A*02:01, A*34:02, and -B*58:02 were overrepresented in the uninfected control group. Our data indicate that HLA alleles may have differential effects against HIV acquisition and disease progression in vertical HIV-1 infection.

Globally, 35.3 million people were estimated to be living with HIV infection in 2012.^1^ Southern Africa remains the worst affected region with adult HIV prevalence rates exceeding 20% in some countries^2^: this rate is even higher among pregnant women.^1^ Without intervention the risk of mother-to-child transmission (MTCT) of HIV is 25–35%, which has resulted in a regional epidemic of vertically acquired HIV, in parallel to the adult epidemic, that has had a major impact on child mortality.^1,3^

Unlike adults, in which the median time from HIV infection to development of AIDS is about 10 years, the natural history of HIV in infants is dominated by high rates of rapid disease progression with a 50% probability of survival by the age of 2 years in African cohorts.^4–6^ As a result, survival into adolescence without antiretroviral treatment was considered rare in Africa. However, recent reports have revealed that up to one-third of HIV-infected infants have more slowly progressive disease with mean survival rates ranging from 8 to 16 years of age.^7–10^

Population-based surveys of older children in Southern Africa report high HIV prevalence rates of 2–6% among older children and young adults.^11^ Although many untreated HIV^+^ adolescents present with severe disease manifestations, predominantly respiratory and cardiac, initial studies suggest that a small proportion (5–10%) of newly diagnosed presumed vertically infected HIV^+^ adolescents in Harare, Zimbabwe have no symptoms and a normal CD4^+^ count,^12^ and could be termed long-term slow-progressors (LTSP). Further studies in the now sizable group of adolescents who have survived long term with vertical HIV-1 infection may provide important information about immune correlates of delayed disease progression following acquisition of HIV infection in infancy.

To date, there have been few studies to identify the genetic determinants of long-term survival following vertical acquisition of HIV-1 infection in African adolescents. The highly polymorphic human leukocyte antigen (HLA) gene network on chromosome 6 is strongly predictive of adult HIV-1 infection outcomes,^13^ with striking associations between HLA alleles and viral control.^14^ HIV-1-infected adults with “protective” HLA class I alleles who go on to exhibit spontaneous viral control show rapid viral clearance in acute HIV-1 infection.^15^ In contrast vertically infected infants have very high peak viral loads (VL) in the first few months of life that decline only slowly^16^: there is no obvious “controller” group in infant HIV-1 infection, raising the question of whether the HLA alleles linked with viral control in adults mediate a similar effect in children.

One previous study in a relatively small population of 61 HIV-infected children in Durban, South Africa showed that slow progression was associated with possession of at least one of three predefined “protective” class I HLA alleles (B*57:03, B*58:01, B*81:01), particularly when these were not shared with the mother^17^: however, these children were not followed into adolescence. A better understanding of the role played by host immunogenetic factors in the long-term control of vertical HIV-1 infection could lead to the design of new therapeutic and preventive strategies for pediatric HIV-1 infection.

We sought to identify the HLA correlates of delayed pediatric HIV-1 disease progression using samples previously collected from a cohort of 123 children and adolescents aged 10–18 years old with (presumed) vertically acquired HIV-1 infection and 276 age-matched uninfected controls recruited from public hospitals and primary health care clinics in Harare, Zimbabwe, as previously described.^12,18^ Vertical HIV infection was assumed to be the most likely source of infection on the basis of criteria developed in Zimbabwe: these include self-report of no sexual debut or blood transfusions, a history of maternal or natural sibling HIV disease/death, and characteristic clinical features of longstanding HIV [≥1 of stunting; history of recurrent minor infections (skin, upper respiratory tract)].

The clinical samples were collected as part of a study focusing on the diagnosis and care of older children with previously undiagnosed HIV-1 infection,^12,18^ hence at recruitment the majority of HIV^+^ participants were not taking antiretroviral therapy (ART). The ideal comparison group would have been untreated vertically infected rapid progressors infected with HIV-1 contemporaneously with the slow progressors: however, these children inevitably had died well before the start of this study. We reasoned that the enrichment of specific HLA alleles in the HIV^+^ group compared to healthy controls would be the result of either increased susceptibility to HIV-1 infection or (more likely) a survival advantage. The study was approved by the Medical Research Council of Zimbabwe (MRCZ), the Biomedical Research and Training Institute (BRTI) IRB, the Harare Hospital Ethics Committee, and the Oxford Tropical Research Ethics Committee (OxTREC). Informed consent/assent was obtained from each participant/guardian, and participants largely belonged to the Shona ethnic group.

HLA class I genotyping was performed by sequencing using locus-specific primers and the BigDye Terminator Cycle Sequencing Ready reaction kit (Applied Biosystems, Foster city, CA) as previously described.^19,20^

Overall, we identified 30 HLA-A, 37 HLA-B, and 26 HLA-C subtypes, with the most frequent being A*30:02 (25.7%), B*53:01 (19.4%), and C*04:01 (25.2%), respectively. Figure 1 depicts the assignment of HLA class I alleles (present at >3% frequency in this population) between the long-term survivors of vertical HIV-1 infection and the uninfected controls. Alleles present at significantly increased frequency in the control population include HLA-A*02:01, -A*34:02, and -B*58:02: of note, HLA-B*58:02 has previously been linked to rapid disease progression in African populations (Zambians and Rwandans).^21^ The only alleles showing significant enrichment in the slow progressors were HLA-C alleles, C*08:02 and C*08:04. There was a trend to enrichment for B*57:03, which is strongly linked with delayed disease progression in African adults,^21,22^ but this did not reach significance in our study (Supplementary Table S1; Supplementary Data are available online at www.liebertpub.com/aid).

**FIG. 1.**
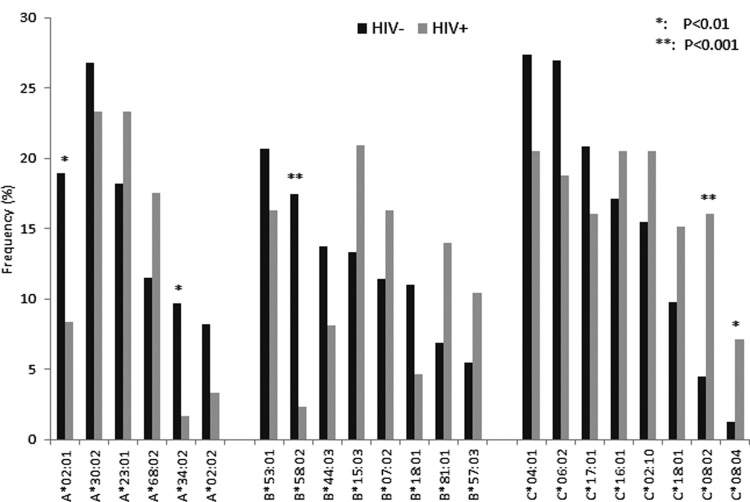
Distribution of HLA class I alleles between infected and uninfected groups. Only alleles with an overall frequency >3% of the study population are represented. *Differences remained significant after Bonferroni correction for multiple comparisons. *p*-values estimated using Chi-squared or Fisher's exact test as appropriate.

These data show that HLA-C*08:02 and C*08:04 are significantly enriched in the HIV-1 long-term survivor group, and may therefore confer a survival advantage following vertical infection (Fig. 1). These alleles have not previously been linked with either progression or disease susceptibility in other cohorts. The level of cell surface expression of some HLA-C alleles is strongly linked with delayed disease progression,^23^ suggesting that HIV-specific T cells restricted by HLA-C molecules may contribute to viral control: however, in this study HLA-C*08:02 was in the middle of the range for HLA-C expression. Alternatively, as ligands for natural killer (NK) cell recognition, HLA-C molecules may contribute to HIV control through NK cell activity: HLA-C*08 alleles belong to the C group 2 allele family, and would therefore be expected to bind to the two domains inhibitory killer immunoglobulin-like receptors (KIRs) 2DL2 and 2DL3. KIR typing and further investigations into potential mechanisms by which HLA-C*08:02 and C*08:04 may contribute to HIV control will be addressed in future studies.

In a large population study, it was shown that HLA-influenced viral polymorphisms correlated with higher viral loads. In the same study, escape mutants were associated with the most prevalent class I allele in most populations, HLA-A*02:01.^24^ This indicates that HLA immune pressure may lead to the evolution of circulating HIV variants that are able to avoid detection by the most common HLA alleles, which, in turn, contributes to the failure of the immune system to control viral replication. In a mixed American population, a strong relationship was found between rapid disease progression in children and the presence of the HLA-A*23:01 allele, but a discordant effect of HLA-A*02:01 alleles in the black and white ethnic groups was noted.^17,25^ In our study, HLA-A*02:01, A*34:02, and HLA-B*58:02 were significantly overrepresented in the uninfected group: this could indicate that these alleles have a beneficial effect against HIV acquisition, but most immunogenetic studies on HIV infection have reported much stronger links between HLA alleles and disease progression than either resistance or susceptibility to HIV acquisition. Alternatively, the relatively low frequencies of these alleles in the long-term survivor group could reflect associations with rapid progression to AIDS and early death following vertical HIV-1 transmission. Further investigation in well-established pediatric cohorts, in which both rapid and slow progressors can be reliably characterized, will be required to resolve this issue.

Host genetic factors have been shown to have a profound impact on antigen presentation and immune responses, greatly influencing HIV pathogenesis and progression to AIDS. The HLA class I region, the most polymorphic region of the human genome, has a stronger association with HIV disease outcome than any other group of genes. Here, we provide the HLA class I gene profiles of a population of adolescent long-term survivors of HIV-1 infection that serve as an essential first step for further study design to elucidate the immunological and genetic factors mediating delayed disease progression in a minority of African children with vertically acquired HIV-1 infection.

## Supplementary Material

Supplemental data
