# Supplementary material for: HLA Correlates of Long-Term Survival in Vertically Infected HIV-1-Positive Adolescents in Harare, Zimbabwe
Source: AIDS Res Hum Retroviruses. 2015 May 1;31(5):504–7. doi: 10.1089/aid.2014.0338 (PMC4426308; doi:10.1089/aid.2014.0338)
Supplement: Supplemental data [file Supp_Table1.pdf]

Supplementary Data

Supplementary Table S1. HLA Class I Genotypes Present in the Study Population

HLA-A	% (n)	*HLA-B*	% (n)	*HLA-C*	% (n)
A*01:01	3.08 (12)	B*07:02	12.83 (39)	C*01:02	0.84 (3)
A*02:01	15.68 (61)	B*08:01	5.26 (16)	C*02:02	3.92 (14)
A*02:02	6.68 (26)	B*13:02	4.28 (13)	C*02:10	17.09 (61)
A*02:05	4.37 (17)	B*14:01	1.97 (6)	C*03:02	3.36 (12)
A*03:01	10.28 (40)	B*14:02	2.63 (8)	C*03:03	1.96 (7)
A*23:01	19.79 (77)	B*15:03	15.46 (47)	C*03:04	8.40 (30)
A*26:01	1.03 (4)	B*15:10	12.50 (38)	C*04:01	25.21 (90)
A*29:01	1.80 (7)	B*15:16	2.30 (7)	C*06:02	24.37 (87)
A*29:02	14.91 (58)	B*18:01	9.21 (28)	C*07:01	19.33 (69)
A*30:01	18.77 (73)	B*35:01	5.59 (17)	C*07:02	9.52 (34)
A*30:02	25.71 (100)	B*39:10	3.62 (11)	C*07:04	7.56 (27)
A*30:04	5.14 (20)	B*40:16	0.99 (3)	C*08:02	8.12 (29)
A*32:01	4.11 (16)	B*41:01	2.63 (8)	C*08:04	3.08 (11)
A*33:01	2.06 (8)	B*42:01	13.82 (42)	C*12:03	3.92 (14)
A*33:03	2.83 (11)	B*42:02	2.30 (7)	C*14:02	1.96 (7)
A*34:02	7.20 (28)	B*44:03	12.17 (37)	C*15:05	1.12 (4)
A*36:01	8.48 (33)	B*45:01	13.16 (40)	C*16:01	18.21 (65)
A*66:01	4.88 (19)	B*49:01	0.99 (3)	C*17:01	19.33 (69)
A*66:03	1.03 (4)	B*51:01	3.62 (11)	C*18:01	11.48 (41)
A*68:01	3.08 (12)	B*53:01	19.41 (59)		
A*68:02	13.37 (52)	B*57:02	1.64 (5)		
A*74:01	13.37 (52)	B*57:03	6.91 (21)		
		B*58:01	9.21 (28)		
		B*58:02	13.16 (40)		
		B*81:01	8.88 (27)		

n, total number of individual carriers of the allele of interest; only genotypes present in approximately 1% of the participants are listed. HLA-A, -B, and -C genotypes data were successfully generated on 389, 304, and 357 participants, respectively.
